# Orthogroup-Based Comparative Analysis of Prophage Gene Content in *Candidatus* Liberibacter Asiaticus Supports a Predominantly Conserved Global Repertoire with Limited Accessory Variation

**DOI:** 10.3390/ijms27125638

**Published:** 2026-06-22

**Authors:** Abdullah F. Alhashel, Ali A. Almasrahi, Mohammed A. Alsaleh, Arya Widyawan, Mahmoud H. El-Komy, Yasser E. Ibrahim

**Affiliations:** Department of Plant Protection, College of Food and Agricultural Sciences, King Saud University, P.O. Box 2460, Riyadh 11451, Saudi Arabiamalsaleh@ksu.edu.sa (M.A.A.); ayanto@ksu.edu.sa (A.W.); malkomy@ksu.edu.sa (M.H.E.-K.)

**Keywords:** Huanglongbing, citrus greening, prophage, OrthoFinder, comparative genomics, pangenome, bacterial genomics

## Abstract

Huanglongbing, a destructive citrus disease of global importance that is also present in Saudi Arabia, is associated with *Candidatus* Liberibacter asiaticus (CLas) and remains a major threat to citrus production. Although previous studies have documented sequence variation and prophage polymorphism in CLas, broader comparisons of prophage-associated gene content remain limited. In particular, comparative orthogroup analysis of prophage gene-content conservation across geographically structured CLas populations has rarely been explored. In this study, we analyzed 42 CLas prophage genomes from Saudi Arabia and other geographic regions using a comparative orthogroup framework. OrthoFinder assigned 99.1% of predicted proteins (1825 of 1841) to 64 orthogroups, with only 16 genes remaining unassigned. A small number of rare orthogroups restricted to only a few genomes were identified, and no orthogroup was detected in all genomes. Presence–absence analyses supported a predominantly conserved prophage gene repertoire together with a small accessory component, while also indicating that apparent absences should be interpreted in light of mixed assembly status and prophage-region completeness. Saudi Arabian genomes were distributed within the broader global framework and exhibited generally similar gene-content profiles rather than a deeply separated lineage. Functional interpretation of representative orthogroups identified conserved prophage-associated genes related to replication, helicase activity, and phage packaging, whereas variable orthogroups were primarily associated with hypothetical or accessory prophage-related functions. Overall, these results are consistent with a model in which CLas prophage diversification is associated more with sequence-level variation and localized structural differences than with extensive gain or loss of prophage genes. These findings further refine current understanding of CLas genome evolution and highlight conserved prophage-associated targets that may support molecular diagnostics and epidemiological surveillance.

## 1. Introduction

Huanglongbing (HLB), also known as citrus greening disease, is widely regarded as one of the most economically important diseases affecting citrus worldwide, including Saudi Arabia [1,2,3,4,5]. Since its spread into major citrus-producing regions, HLB has caused severe economic losses through reduced yield, poor fruit quality, increased management costs, and progressive tree decline [1,4].

HLB is most commonly associated with phloem-limited, Gram-negative bacteria belonging to the α-subdivision of the Proteobacteria, collectively classified within the genus *Candidatus* Liberibacter [6]. Among these species, *Candidatus* Liberibacter asiaticus (CLas) is the most widespread and economically important in commercial citrus production. It is transmitted primarily by the Asian citrus psyllid, *Diaphorina citri* [7,8,9].

Genome sequencing has shown that CLas possesses a small, reduced genome with features consistent with adaptation to a host-dependent, phloem-restricted lifestyle [10,11]. In host-associated bacteria, genome reduction is often accompanied by loss of metabolic functions, increased reliance on host resources, and fewer accessory capabilities than in free-living species [12]. Although the CLas genome is streamlined, several regions, particularly prophages and other mobile DNA-associated loci, appear to contribute disproportionately to its genetic diversity [13,14].

Among these variable regions, prophage-associated sequences are of particular interest because they are linked to recombination, structural polymorphism, and lineage differentiation. Previous studies identified multiple prophage types in CLas, including SC1- and SC2-like elements, and demonstrated their usefulness for population studies, phylogenetic inference, and comparative genomics [13,14]. In addition to their utility for genotype differentiation and population tracking, prophage-associated regions may contribute to genome plasticity, localized structural diversification, and recombination within CLas populations [13,14,15,16]. Because prophage-associated loci frequently exhibit greater variability than conserved housekeeping regions, they have become useful targets for epidemiological surveillance and comparative genomic studies [14,15,16]. Earlier studies also detected geographic structure in CLas populations, while more recent analyses based on larger genome collections supported substantial overall genomic similarity among analyzed isolates [15,16].

Although nucleotide-based analyses have substantially improved our understanding of CLas evolution, sequence polymorphism alone does not fully explain how genomes diversify. Two isolates may differ by single nucleotide polymorphisms (SNPs) while retaining nearly identical gene repertoires, whereas others may differ through gain, loss, or differential retention of specific loci. Orthogroup-based comparative analysis helps address this limitation by clustering homologous proteins into shared evolutionary units, allowing evaluation of conserved, accessory, and lineage-restricted gene content across multiple genomes [17,18]. Such analyses can help distinguish stable prophage-associated functions from variable loci potentially involved in recombination, regulation, or adaptation. This approach has been widely used in bacterial comparative genomics but has only rarely been applied to CLas prophage regions, where earlier studies focused primarily on sequence polymorphism and prophage typing rather than broader gene-content comparisons [13,14].

Because prophage-associated regions often represent dynamic components of bacterial genomes, understanding whether their variation reflects extensive gene turnover or predominantly sequence-level diversification may improve interpretation of CLas population structure and epidemiological relationships. Comparative analysis of prophage gene content may also help identify conserved loci suitable for molecular diagnostics together with variable regions useful for population tracking and surveillance.

Saudi Arabia provides a particularly relevant yet understudied setting for this question. Citrus production in the Arabian Peninsula occurs under hot and arid conditions that differ markedly from many long-established HLB-endemic regions. Recent nationwide surveys confirmed the presence and distribution of CLas in several citrus-growing areas of Saudi Arabia, while subsequent genomic analyses suggested that many Saudi isolates form a relatively cohesive population with limited internal divergence [5,19]. However, it remains unclear whether this apparent cohesion also extends to prophage gene-content organization.

Accordingly, the present study evaluated conservation and variation in prophage-associated gene content across Saudi Arabian and globally distributed CLas genomes using a comparative orthogroup framework. Specifically, we aimed to (i) quantify shared, accessory, and lineage-restricted prophage orthogroups; (ii) assess whether apparent gene-content differences reflect broad genome-level divergence or limited variation in specific accessory components; and (iii) determine whether Saudi Arabian isolates form a distinct prophage gene-content profile or remain embedded within the broader global CLas prophage framework.

## 2. Results

### 2.1. Overall Orthogroup Conservation and Accessory Diversity

**Figure 1:**
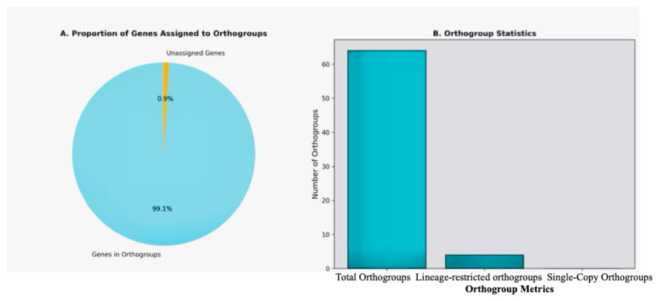
Summary of orthogroup assignment across the 42 analyzed *Candidatus* Liberibacter asiaticus prophage genomes. The pie chart (**A**) shows that 1825 of 1841 predicted proteins (99.1%) were assigned to orthogroups, whereas only 16 proteins (0.9%) remained unassigned. The bar chart (**B**) summarizes orthogroup structure, highlighting the total number of identified orthogroups and the small subset of lineage-restricted orthogroups. Together, these results indicate a highly conserved prophage gene repertoire with limited accessory diversity.

Across the 42 analyzed genomes, a total of 1841 predicted proteins encoded within prophage-associated regions were included in the orthology analysis. Of these, 1825 proteins were assigned to orthogroups, corresponding to an assignment rate of 99.1%, whereas only 16 proteins (0.9%) remained unassigned. In total, 64 orthogroups were identified, including four lineage-restricted orthogroups comprising eight genes (Figure 1). Orthogroup assignment efficiency remained high across both complete and draft genome assemblies, suggesting that assembly status had limited influence on overall orthogroup recovery. The high assignment efficiency indicates that most prophage-encoded proteins retained detectable homologous relationships under the OrthoFinder workflow and supports the presence of a compact comparative gene repertoire. Despite this high assignment rate, orthogroup sharing was not uniform across all genomes, and no orthogroup was detected in every analyzed genome, probably reflecting a combination of true prophage-type variation, partial prophage recovery, annotation differences, and assembly fragmentation. The small number of lineage-restricted orthogroups indicates that unique diversity was limited. Relative to the total number of analyzed proteins and orthogroups, these findings show that accessory diversity exists but represents only a minor fraction of the total prophage gene repertoire (Figure 1).

### 2.2. Genome-Level Gene-Count Distributions Are Relatively Stable

**Figure 2:**
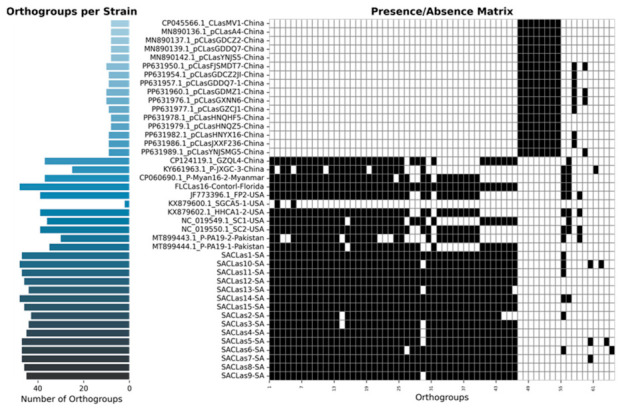
Comparative presence–absence matrix of orthogroup distribution across the 42 analyzed *Candidatus* Liberibacter asiaticus prophage genomes. The left horizontal bars indicate the total number of orthogroups detected in each genome, showing generally similar gene-content profiles with moderate variation among isolates. The matrix on the right displays binary orthogroup presence–absence patterns across genomes, where filled cells indicate presence (black dots) and empty cells indicate absence. Genomes are shown in comparative display order rather than hierarchical clustering order. Broadly distributed orthogroups form the dominant conserved prophage-associated framework, whereas a smaller subset of sparse patterns represents accessory or lineage-restricted variation concentrated within a limited number of orthogroups.

The number of orthogroup-associated genes per genome showed moderate variation among isolates. Orthogroup-associated gene counts ranged from 38 to 48 genes across most analyzed prophage-associated regions. Much of the observed variation reflected differences in prophage-region completeness, draft assembly fragmentation, and partial prophage recovery rather than major biological divergence. Most genomes nevertheless exhibited broadly similar orthogroup assignment patterns, whereas only a limited subset showed reduced totals associated mainly with fragmented or partial assemblies. The full genome-level distribution is shown in Supplementary Figure S1. No genomes displayed evidence of marked lineage-specific expansion or large-scale contraction of prophage-associated gene inventories. These patterns indicate that differences among isolates were quantitative and modest rather than reflective of major structural divergence (Figure 2). The generally uniform orthogroup counts across genomes further suggest that prophage-associated regions in CLas retain a relatively stable functional organization despite geographic separation among isolates.

### 2.3. Presence–Absence Patterns Support a Dominant Conserved Framework

Binary presence–absence comparisons across the 64 orthogroups revealed that most orthogroups were broadly distributed among genomes, forming the dominant comparative framework of the dataset. A smaller number of orthogroups showed patchy occurrence or restriction to subsets of isolates, consistent with accessory variation. Overall, the matrix pattern supports extensive shared gene content accompanied by limited heterogeneity concentrated in a relatively small number of orthogroups (Figure 2). To facilitate biological interpretation of orthogroup distribution patterns, representative conserved and variably distributed orthogroups together with their annotations and comparative distribution patterns across analyzed genomes are summarized in Supplementary Table S3.

### 2.4. Clustering and Lineage-Restricted Variation

**Figure 3:**
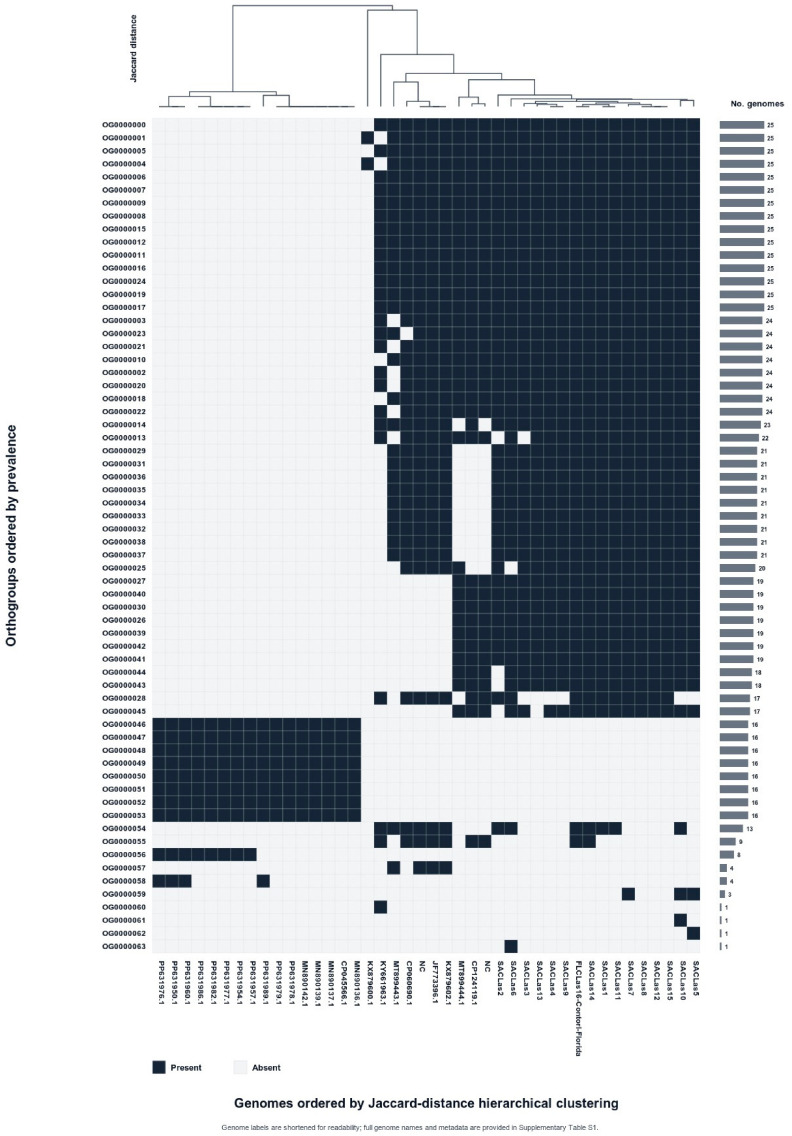
Hierarchically clustered heatmap of orthogroup presence–absence across 42 analyzed *Candidatus* Liberibacter asiaticus prophage genomes. Rows represent orthogroups and columns represent genomes reordered according to hierarchical clustering of binary orthogroup presence–absence profiles. Dark cells indicate presence and light cells indicate absence. The dendrogram summarizes relative similarity relationships in prophage gene-content organization among genomes rather than strict phylogenetic relatedness. Saudi Arabian isolates are distributed within the broader global comparative structure and exhibit generally similar orthogroup profiles within a predominantly conserved prophage-associated gene repertoire.

Although Figure 2 suggests broad similarity groupings associated with differences in accessory orthogroup composition, the clustering structure recovered in Figure 3 remained relatively diffuse. This pattern is consistent with the high degree of shared orthogroup content observed across the dataset and indicates that most genomes retain broadly similar prophage-associated gene repertoires despite localized accessory variation. Saudi Arabian genomes were distributed within the broader global comparative structure and exhibited generally similar orthogroup profiles rather than forming a deeply divergent lineage. Accordingly, the observed clustering patterns are best interpreted as modest similarity relationships within a largely conserved prophage-associated gene-content framework rather than evidence of strong geographic separation or strict phylogenetic subdivision. Details of lineage-restricted orthogroups together with representative annotations and comparative genome distribution patterns are provided in Supplementary Table S3. Representative conserved orthogroups were primarily associated with replication/helicase-related proteins and prophage packaging functions, whereas variably distributed orthogroups were more commonly associated with hypothetical or accessory prophage-related proteins. These patterns further support the presence of a conserved prophage-associated functional backbone together with limited accessory variation across analyzed genomes.

### 2.5. Orthogroup Frequency Spectrum Supports a Predominantly Conserved Repertoire

**Figure 4:**
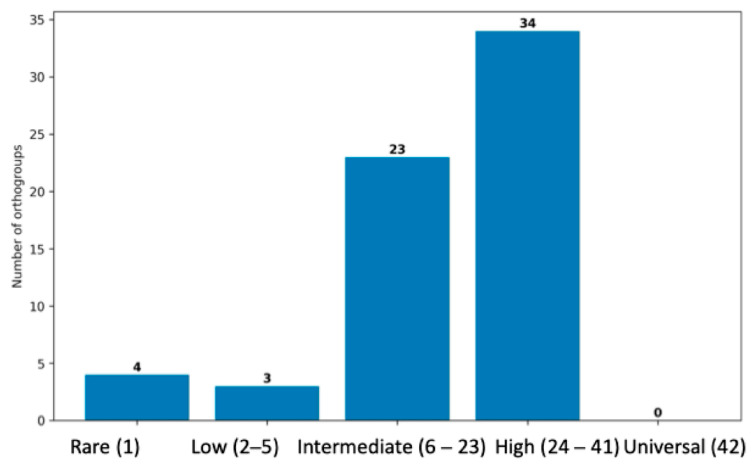
Frequency distribution of orthogroups across 42 analyzed *Candidatus* Liberibacter asiaticus prophage genomes. Bars indicate the number of orthogroups detected within each genome-frequency class. Most orthogroups were broadly distributed across multiple genomes, supporting the presence of a conserved prophage-associated gene repertoire. In contrast, only a small subset of orthogroups occurred infrequently or in lineage-restricted patterns, representing a limited accessory component. No orthogroup was detected in all analyzed genomes.

Frequency analysis of orthogroup occurrence across the 42 analyzed genomes revealed that most orthogroups were broadly distributed, with 34 orthogroups present in 21–41 genomes and 23 present in 6–20 genomes (Figure 4). Only seven orthogroups occurred in five or fewer genomes, including four restricted to a single genome. No orthogroup was detected in all 42 genomes. These findings indicate that CLas prophage diversity is dominated by broadly distributed orthogroups, whereas rare lineage-restricted components represent only a minor fraction of the repertoire. The observed frequency distribution suggests that variation within CLas prophage-associated regions is concentrated primarily within a limited accessory component rather than the conserved functional backbone. The absence of universally shared orthogroups likely reflects localized variation within a small subset of prophage-associated loci together with sequence divergence and partial assembly fragmentation in some genomes (Figure 4).

### 2.6. Saudi Arabian Genomes Fall Within the Global Conserved Structure

Saudi Arabian genomes showed comparatively uniform orthogroup profiles and were positioned within the same broad conserved structure observed across the global dataset. Their gene-count distributions did not indicate major expansion or reduction relative to non-Saudi genomes. Likewise, presence–absence patterns did not reveal a distinct large-scale restructuring of prophage-associated gene content. Instead, Saudi isolates were characterized by limited divergence within an otherwise shared global framework (Figure 2 and Figure 3; Supplementary Table S1). Pairwise Jaccard similarity analysis based on orthogroup presence–absence profiles further supported the relatively conserved structure of Saudi Arabian prophage-associated genomes. Saudi-versus-Saudi comparisons exhibited consistently high similarity values, whereas comparisons involving non-Saudi genomes were more heterogeneous. This broader variability likely reflects differences in prophage composition, sequence divergence, prophage-region completeness, and mixed assembly status across globally distributed genomes (Supplementary Table S4).

### 2.7. Integrated Summary of Orthogroup Patterns

Taken together, orthogroup assignment rates, gene-count distributions, lineage-restricted orthogroups, and binary presence–absence patterns converge on a consistent interpretation: CLas prophage genomes are dominated by a conserved gene-content backbone with only modest accessory variation. Observed diversity among lineages appears limited in magnitude and is not primarily driven by extensive gain or loss of prophage genes.

## 3. Discussion

This orthogroup-based analysis supports the interpretation that prophage-associated gene content in CLas is predominantly conserved across geographically distinct populations. The assignment of 99.1% of predicted proteins to orthogroups indicates that most prophage genes have identifiable homologs across the dataset, supporting the interpretation of a compact and evolutionarily constrained gene repertoire. High orthogroup assignment efficiency is generally consistent with extensive sequence conservation and limited gene-content turnover, particularly in host-associated bacterial systems characterized by restricted ecological niches [12,18,20].

The functional composition of orthogroups provides additional biological insight into this conserved structure. Several orthogroups correspond to core prophage functions, including DNA replication (e.g., primase and helicase), nucleotide metabolism, and phage packaging (e.g., terminase subunits). These conserved elements likely represent the essential functional backbone required for prophage maintenance and replication. In contrast, a smaller subset of orthogroups exhibited variable distribution and included genes associated with recombination, transcriptional regulation, and putative anti-repressor functions. This partitioning into conserved and variable components suggests that while the core prophage machinery is maintained across CLas populations, limited flexibility is retained in accessory loci that may modulate prophage behavior under specific conditions. Because many prophage-associated proteins remain highly divergent and poorly characterized, functional assignments in the present study should be interpreted as broad functional categories rather than definitive protein-function predictions. More sensitive phage-oriented annotation strategies, including structure-based and profile-based approaches, may further refine characterization of lineage-variable orthogroups in future studies. These observations further support a model of constrained prophage evolution in CLas, in which core functional elements are conserved while accessory variation remains limited.

The representative orthogroups summarized in Supplementary Table S3 further illustrate the predominance of conserved prophage-associated functional categories across geographically distributed CLas populations. Broadly distributed orthogroups were primarily associated with replication-, helicase-, and packaging-related prophage functions, suggesting conservation of core prophage maintenance machinery across analyzed genomes. In contrast, variably distributed orthogroups were more commonly associated with hypothetical or accessory prophage-related proteins, indicating that accessory diversification within CLas prophage-associated regions remains limited and concentrated within a relatively small subset of loci. The predominance of hypothetical proteins among lineage-variable orthogroups also highlights the current limitations of prophage functional annotation and suggests that additional high-sensitivity comparative and structure-based analyses may further refine characterization of these accessory components in future studies.

The reduced genome architecture of CLas, characterized by limited metabolic capacity and strong dependence on host-derived resources, has been recognized since the earliest genome assemblies [10,11]. Such features are typical of obligately host-associated bacteria and are often associated with reduced rates of horizontal gene acquisition compared with free-living species. Our findings extend this concept by showing that even prophage-associated regions—typically among the most dynamic components of bacterial genomes—remain comparatively constrained in gene-content organization within CLas populations. Although prophages can facilitate recombination, structural polymorphism, and adaptive diversification in many bacterial systems, their contribution to large-scale gene-content variability in CLas appears limited [13,21]. Available evidence further suggests that major prophage types in CLas are predominantly maintained through vertical inheritance within established lineages, although localized recombination and structural diversification have also been reported in previous comparative genomic analyses [13,14,15,16].

Although no orthogroup was detected in all genomes, this observation should be interpreted cautiously. Apparent absences may reflect assembly fragmentation, annotation inconsistencies, or sequence divergence below clustering thresholds rather than true gene loss. This limitation is particularly relevant when comparing a mixture of complete and draft genomes derived from different sequencing strategies. Nevertheless, the very small number of lineage-restricted orthogroups indicates that novel gene-content variation is quantitatively minor. Because the analyzed dataset included both complete and fragmented prophage-associated assemblies, some apparent orthogroup absences could not always be distinguished unequivocally between biological deletions and assembly-related fragmentation effects. These observations are consistent with previous studies suggesting that much of the observed CLas diversity is associated primarily with sequence-level variation, prophage sequence types, and localized structural polymorphism rather than extensive gain or loss of prophage-associated genes [14,15,16].

The clustering analysis further refines this interpretation by showing that Saudi Arabian genomes are distributed within the broader global similarity structure while maintaining generally similar orthogroup profiles. Their profiles support earlier reports describing Saudi populations as genetically coherent based on prophage and whole-genome analyses [5,19]. Importantly, this pattern is observed not only at the level of nucleotide variation but also in gene-content structure, indicating that Saudi isolates share a largely conserved prophage architecture. However, these genomes are not separated by deep or exclusive branching, suggesting that their distinctiveness is modest and likely reflects recent population history, ecological filtering under arid production systems, or sequence-level divergence superimposed on a shared genomic framework.

The orthogroup frequency spectrum further supports the predominance of a conserved prophage-associated gene repertoire. Most orthogroups were distributed across multiple genomes, whereas only a small subset occurred rarely or in lineage-restricted patterns. This distribution is consistent with constrained pangenome dynamics commonly observed in specialized host-associated bacteria, where functional requirements limit extensive gene acquisition and loss [17,22].

From an applied perspective, the high level of conservation identified in this study has important implications for disease diagnostics and epidemiological surveillance. Conserved prophage-associated genes may provide candidate molecular targets that complement existing qPCR-based detection methods, particularly in contexts requiring broad geographic applicability. At the same time, the small set of variably distributed orthogroups identified here represents a potential source of markers for population differentiation and tracking. Although most lineage-restricted genes were annotated as hypothetical proteins, their restricted distribution suggests that they may play roles in host interaction, persistence in planta, vector association, or adaptation to local environmental conditions. These hypotheses require experimental validation but highlight the value of integrating gene-content analysis with functional and ecological studies. Overall, the results support a model in which CLas diversification occurs primarily through sequence-level variation within a largely conserved prophage-associated gene repertoire. Taken together, orthogroup assignment rates, gene-count distributions, lineage-restricted orthogroups, and binary presence–absence patterns consistently support the presence of a highly conserved prophage gene-content backbone with only modest accessory variation across analyzed genomes. These findings refine current understanding of CLas population biology and provide a comparative framework for future studies linking prophage-associated variation with epidemiological behavior, diagnostics, and host–pathogen interactions.

A remaining limitation is that the present analysis did not formally resolve syntenic deletion patterns across all prophage-associated regions. Because the dataset included complete prophage sequences together with prophage-associated regions derived from draft assemblies and fragmented contigs, apparent orthogroup absences could not always be distinguished unequivocally from incomplete recovery of the corresponding loci. Future analyses using complete or near-complete prophage assemblies combined with comparative synteny visualization tools such as clinker and high-sensitivity annotation approaches, including HHpred or structure-based comparisons, may provide stronger evidence for distinguishing true structural deletions from technical absence calls.

## 4. Materials and Methods

### 4.1. Genome Dataset and Sampling Framework

A total of 42 CLas prophage genomes were analyzed in this study. The dataset included 15 Saudi Arabian genomes generated previously from infected citrus samples and 27 non-Saudi genomes representing China, the United States, Pakistan, Myanmar, Brazil, and other publicly available sources. Public genome assemblies were retrieved from the NCBI GenBank database. Metadata, accession numbers, geographic origin, and sequence or assembly type information for all analyzed prophage genome sequences are provided in Supplementary Table S1. Both complete prophage genome sequences and prophage-associated regions derived from draft genome assemblies were included when sufficient sequence continuity and identifiable prophage-associated loci were available for comparative analysis.

### 4.2. Definition and Extraction of Prophage-Associated Regions

Comparative analyses were restricted to prophage-associated regions because these loci represent important sources of genomic variability in CLas [13,14]. Previously characterized SC1- and SC2-like prophage architectures were used as reference templates to identify homologous regions across genomes. Where annotations were available, prophage boundaries were guided by annotated prophage genes and conserved syntenic context. For draft assemblies, homologous contigs or regions containing prophage-associated genes were identified using sequence similarity searches and gene-content correspondence relative to the reference prophage regions. This standardized framework was used to maximize comparability among genomes generated from different sources. Regions were retained when they contained prophage-associated coding sequences showing sequence similarity to reference prophage genes and conserved gene-content context. For draft assemblies, contigs were included only when they contained identifiable prophage-associated genes and sufficient continuity to support assignment. Highly fragmented or ambiguous regions lacking clear prophage-associated features were excluded from downstream analysis.

### 4.3. Gene Prediction and Protein Sequence Preparation

Protein-coding sequences within defined prophage regions were obtained from existing GenBank annotations when available. Where standardized re-annotation was required, coding sequences were predicted using Prokka v1.14 under default bacterial annotation settings [23]. The resulting protein FASTA files were manually checked for formatting consistency before orthology analysis. When necessary, sequence identity searches against public databases were used to confirm expected annotation identity. Functional annotation of representative orthogroups was based on Prokka-generated annotations together with available GenBank annotations and was used primarily for broad functional categorization (e.g., replication-, recombination-, packaging-, and hypothetical protein-associated functions) rather than detailed protein-function prediction.

### 4.4. Orthogroup Inference

Orthologous gene groups were inferred using OrthoFinder v2.5 [18]. OrthoFinder performs all-versus-all sequence similarity searches, infers graph-based relationships among proteins, and clusters homologous genes into orthogroups. The analysis was conducted using the standard OrthoFinder workflow with DIAMOND-based protein similarity searches [24]. No additional user-defined identity or coverage thresholds beyond the default OrthoFinder workflow were imposed. Primary outputs used in this study included Orthogroups.tsv, Orthogroups. GeneCount.tsv, and summary statistics files. Because the analyzed dataset included both complete and fragmented prophage-associated assemblies, apparent orthogroup absences were interpreted cautiously in the context of possible assembly fragmentation, partial coding sequences, and annotation variability. For transparency and reproducibility, software versions and primary workflow components used in the analysis are reported herein.

### 4.5. Presence–Absence Matrix Construction

To compare orthogroup distribution among genomes, OrthoFinder gene-count matrices were converted to binary presence–absence matrices following commonly used comparative genomics approaches [17]. Any orthogroup with a gene count greater than zero in a genome was coded as present (1), whereas zero counts were coded as absent (0). Because the analyzed dataset included both complete prophage sequences and prophage-associated regions derived from draft assemblies, apparent orthogroup absences were interpreted conservatively and were not considered definitive deletions unless supported by assembly continuity and regional genomic context. Hierarchical clustering of the binary presence–absence matrix was performed using Jaccard distance and average linkage to visualize relative similarity relationships among genomes. Orthogroup frequency spectra were calculated by counting the number of genomes in which each orthogroup was present and grouping orthogroups into predefined occurrence classes: presence in 1 genome, 2–5 genomes, 6–20 genomes, 21–41 genomes, or all 42 genomes. Pairwise similarity among genomes was additionally evaluated using Jaccard distance based on binary orthogroup presence–absence data to support descriptive interpretation of clustering patterns. Summary pairwise similarity statistics for Saudi-versus-Saudi, Saudi-versus-non-Saudi, and non-Saudi-versus-non-Saudi genome comparisons were calculated from the binary orthogroup presence–absence matrix.

### 4.6. Comparative Interpretation of Gene-Count Distributions

The total number of orthogroup-associated genes per genome was summarized from OrthoFinder gene-count outputs. A genome-by-genome visualization of these distributions is provided in Supplementary Figure S1. Because the analyzed genomes originated from multiple sources and may differ in assembly completeness, comparisons were interpreted descriptively rather than as formal tests of differential genome content. Emphasis was placed on overall distribution patterns, relative uniformity, and evidence for major expansion or reduction events.

### 4.7. Data Visualization and Software Environment

Summary graphics, bar plots, heatmaps, and matrix-style visualizations were generated using standard scientific computing environments in R version 4.1.0 (ggplot2 package) and Python (Matplotlib 3.11.0). Figures were designed to illustrate orthogroup assignment efficiency, genome-level gene-count distributions, clustering relationships, and presence–absence structure across the 42 analyzed genomes. Analyses emphasized biological interpretation of comparative patterns rather than inferential statistics because of unequal geographic representation and heterogeneous source assemblies.

### 4.8. Reproducibility Considerations

All analyses were based on archived genome assemblies and deterministic software workflows using fixed input datasets. Because orthogroup inference can be influenced by annotation quality and assembly fragmentation, outputs were interpreted in the context of known limitations of draft genomes. Nevertheless, the high proportion of assigned proteins supports the robustness of the overall comparative framework.

## 5. Conclusions

Orthogroup-based comparison of 42 CLas prophage genomes supports the presence of a predominantly conserved global prophage-associated gene repertoire with modest accessory variation. The high orthogroup assignment rate indicates extensive detectable homology among prophage-encoded proteins, although the absence of universally detected orthogroups across all genomes highlights the importance of cautious interpretation of apparent absence calls in datasets containing mixed assembly types and fragmented prophage-associated regions. Saudi Arabian genomes were distributed within the broader global comparative framework and exhibited generally similar orthogroup profiles rather than forming a deeply divergent lineage. Collectively, these findings support a model in which diversification of CLas prophage-associated regions is limited in magnitude and occurs primarily through sequence-level variation and localized structural polymorphism rather than extensive gain or loss of prophage-associated genes. The present study provides a comparative framework for future genomic surveillance, diagnostic marker development, and functional investigation of conserved and variably distributed prophage-associated loci.

## Data Availability

All genome assemblies analyzed in this study are publicly available through NCBI GenBank under the accession numbers listed in Supplementary Table S1. Processed summary datasets generated during this study are available from the corresponding author upon reasonable request.

## Supplementary Materials

The following supporting information can be downloaded at: https://www.mdpi.com/article/10.3390/ijms27125638/s1.
