# Supplementary material for: Orthogroup-Based Comparative Analysis of Prophage Gene Content in Candidatus Liberibacter Asiaticus Supports a Predominantly Conserved Global Repertoire with Limited Accessory Variation
Source: Int J Mol Sci. 2026 Jun 22;27(12):5638. doi: 10.3390/ijms27125638 (PMC13299691; doi:10.3390/ijms27125638)
Supplement: Supplementary file 1 [file ijms-27-05638-s001.zip › ijms-4321730-supplementary.pdf]

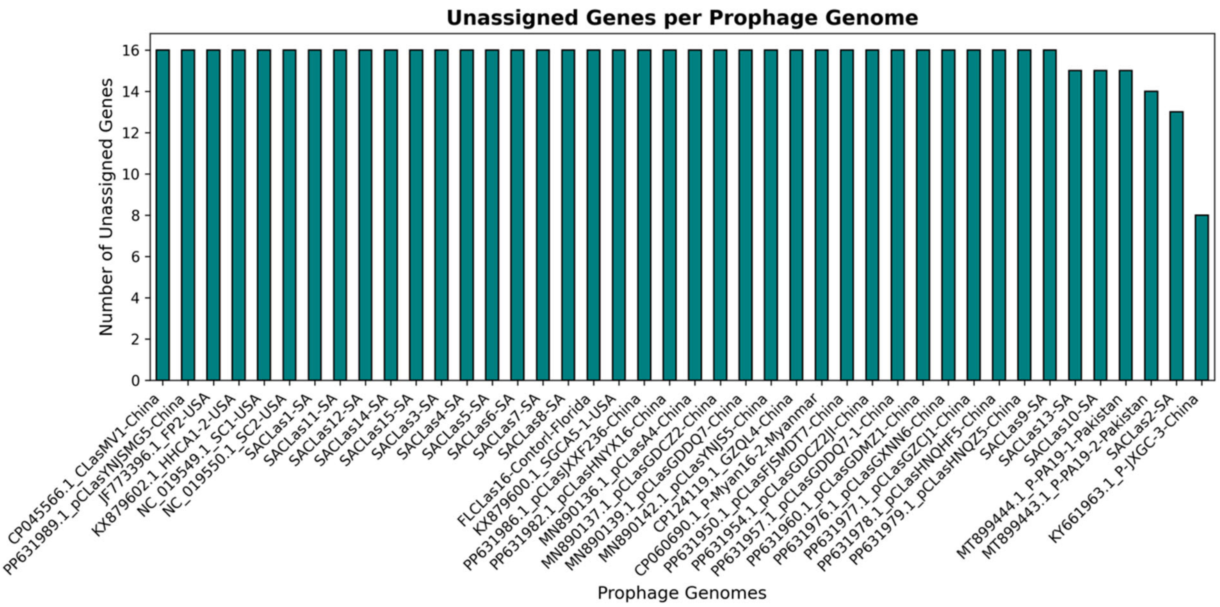

Supplementary Figure S1. Distribution of orthogroup-associated gene counts across the 42 analyzed *Candidatus* Liberibacter asiaticus prophage genomes. Each bar represents the total number of genes assigned to orthogroups in an individual genome. Most genomes show similar counts, indicating broadly stable prophage gene-content organization, whereas a small number of isolates exhibit reduced totals.

Supplementary Table S1. Metadata and geographic origin of the 42 Candidatus Liberibacter asiaticus genome assemblies analyzed in this study.

Genome ID	Country/Region	Group	Source
SACLas1	Saudi Arabia	Saudi	Ibrahim et al.2022
SACLas2	Saudi Arabia	Saudi	Ibrahim et al.2022
SACLas3	Saudi Arabia	Saudi	Ibrahim et al.2022
SACLas4	Saudi Arabia	Saudi	Ibrahim et al.2022
SACLas5	Saudi Arabia	Saudi	Ibrahim et al.2022
SACLas6	Saudi Arabia	Saudi	Ibrahim et al.2022
SACLas7	Saudi Arabia	Saudi	Ibrahim et al.2022
SACLas8	Saudi Arabia	Saudi	Ibrahim et al.2022
SACLas9	Saudi Arabia	Saudi	Ibrahim et al.2022
SACLas10	Saudi Arabia	Saudi	Ibrahim et al.2022
SACLas11	Saudi Arabia	Saudi	Ibrahim et al.2022
SACLas12	Saudi Arabia	Saudi	Ibrahim et al.2022
SACLas13	Saudi Arabia	Saudi	Ibrahim et al.2022
SACLas14	Saudi Arabia	Saudi	Ibrahim et al.2022
SACLas15	Saudi Arabia	Saudi	Ibrahim et al.2022
CLasMV1	China	Non-Saudi	CP045566.1
GZQL4	China	Non-Saudi	CP124119.1
P-JXGC-3	China	Non-Saudi	KY661963.1
pCLasA4	China	Non-Saudi	MN890136.1
pCLasFJSMDT7	China	Non-Saudi	PP631950.1
pCLasGDCZ2	China	Non-Saudi	MN890137.1
pCLasGDCZ2JI	China	Non-Saudi	PP631954.1
pCLasGDDQ7	China	Non-Saudi	MN890139.1
pCLasGDDQ7-1	China	Non-Saudi	PP631957.1
pCLasGDMZ1	China	Non-Saudi	PP631960.1

pCLasGXNN6	China	Non-Saudi	PP631976.1
pCLasGZCJ1	China	Non-Saudi	PP631977
pCLasHNQHF5	China	Non-Saudi	PP631978
pCLasHNQZ5	China	Non-Saudi	PP631979.1
pCLasHNYX16	China	Non-Saudi	PP631982.1
pCLasJXXF236	China	Non-Saudi	PP631986.1
pCLasYNJS5	China	Non-Saudi	MN890142.1
pCLasYNJSMG5	China	Non-Saudi	PP631989.1
FP2	USA	Non-Saudi	NC_021042.1
HHCA1-2	USA	Non-Saudi	KX879602.1
SC1	USA	Non-Saudi	NZ_BBRO01000001.1
SC2	USA	Non-Saudi	NM_139579
SGCA5-1	USA	Non-Saudi	KX879601.1
FLCLas16-Control	USA (Florida)	Non-Saudi	XM_059788054
P-PA19-1	Pakistan	Non-Saudi	MT899444.1
P-PA19-2	Pakistan	Non-Saudi	MT899443.1
P-Myan16-2	Myanmar	Non-Saudi	CP060690.1

Supplementary Table S2. Lineage-restricted orthogroups identified in CLas prophage genomes

**Genome ID**	**No. of lineage-restricted orthogroups**	**No. of genes**	**Representative gene annotation***	**Interpretation**
SACLas3	1	2	hypothetical / prophage-associated protein	Saudi lineage-restricted
SACLas4	1	2	hypothetical / prophage-associated protein	Saudi lineage-restricted
pCLasJXXF236	1	2	hypothetical / prophage-associated protein	non-Saudi lineage-restricted

*Annotations are based on available Prokka/GenBank functional predictions; most lineage-restricted genes were annotated as hypothetical or prophage-associated proteins.
